# Credence in the Organization’s Ability to Respond to Change – Implications on Work Engagement and Job Satisfaction in the Church of Sweden

**DOI:** 10.3389/fpsyg.2020.00995

**Published:** 2020-06-12

**Authors:** Anders Edvik, Martin Geisler, Tuija Muhonen, Hope Witmer, Josefin Björk

**Affiliations:** Centre for Work Life and Evaluation Studies, Malmö University, Malmö, Sweden

**Keywords:** religious organizations, change, credence, job satisfaction, work engagement

## Abstract

As part of society, religious organizations are exposed to contextual conditions and challenges. However, adapting to external conditions is an act of balance since too much compromising may risk having a negative effect on employees’ perception of organizational authenticity and, in turn, employees’ well-being and attitudes toward work. In this study, we examined how specific characteristics of the work, in terms of job demands (role conflict and emotional demands) and job resources (influence at work and social community at work), as well as employees’ credence in the organization’s ability to respond to change, relate to employee well-being within the Church of Sweden. In total 2,112 employees (58% participation rate) answered a web-based survey. The results of regression analyses showed that job resources and credence in the organization’s ability to respond to change provided a clear contribution to the explanation of variance in work engagement and, especially, job satisfaction. However, the contribution of job demands was less clear. Moreover, to further the understanding of the association between employees’ credence in the organization’s ability to respond to change and employee well-being, the mediating effect of job resources was tested. The results showed that the association between credence and well-being is in part mediated by job resources. In sum, the study demonstrate that employees’ credence in the organization’s ability to respond to change is important to consider for understanding employee well-being within religious organizations. In conclusion, our study suggest that organizations that are built up on strong values and institutionalized beliefs, such as religious and faith-based organizations, need to tread carefully in the process of adapting to conformal pressure for change. This, since the actions and choices of the organization are important for employees’ credence in the organization and, in turn, employee well-being. Implications and recommendations for future research are discussed.

## Introduction

In recent years, increased attention has been paid to the societal importance of religious organizations as well as the well-being of the employees in these organizations (e.g., Roof, 2015; Ariza-Montes et al., 2017). As provider of a wide range of welfare services (e.g., social care, daycare, deaconry, and elderly care), religious organizations not only give believers a community to belong to, but they also are subject to societal challenges. Hence, as part of society, religious organizations are not bound to one institutionalized community but exposed to external challenges, expectations, and norms other than merely religious ones (DiMaggio and Powell, 1983; Suchman, 1995; Deephouse and Suchman, 2008). These logics and rationalities of organizational structures, management conceptions, and operational standards constitute a conformal pressure on the organization to adapt to (e.g., Brunsson and Jacobsson, 2010; Brunsson et al., 2012). However, religious organizations are based on beliefs and collectively shared traditions, often expressed through ceremonies that in turn shape identity, meaning, and engagement for employees (e.g., Roof, 2015). Furthermore, even the organizational structure of religious organizations carries a congregational heritage (e.g., spirituality and authenticity), which influences working conditions as well as employees’ attitudes toward work (Roof, 2015; Ariza-Montes et al., 2017). Thus, initiating organizational adjustments to meet external pressure in ways that compromise or neglect core values may have negative effects on employees’ well-being. Moreover, the associations seem to be complex, as the ability of organizations to handle change in a constructive manner is dependent on the commitment of the employees (Elias, 2009).

Based on these considerations, the aim of the present study was to investigate the role and importance of credence in the organization’s ability to respond to changes in relation to employee well-being in religious organizations. Specifically, in a larger sample of employees of the Church of Sweden, we investigated how specific job characteristics (i.e., job demands and job resources), and employees’ credence in the organization’s ability to respond to contextual changes, relate to job satisfaction and work engagement.

### Job Demand and Resource Theory

Working conditions and employees’ well-being are major concerns for the strategic and long-term performance of organizations (Bakker, 2017). Research on the Job Demands–Resources (JD-R) theory suggests that job demands and job resources explain motivational and/or health-impairment processes (e.g., Schaufeli and Bakker, 2004; Bakker and Demerouti, 2017, 2018). Job resources foster intrinsic motivation through sense of belonging, competence, and autonomy, and contribute to higher levels of meaning and responsibility in work. When the levels of resources are higher, it is more likely that individuals experience higher job satisfaction, work engagement, and commitment to work. However, the opposite applies at higher levels of demands and lower levels of resources. According to research, the balance between job demands and resources explains the motivational and the health-impairment processes, which in turn determines employees’ occupational health and well-being (e.g., Bakker and Demerouti, 2017, 2018; Lesener et al., 2019). In occupations with higher levels of demands, such as contradictory and emotional demands, there is an obvious risk of lower levels of employee well-being, higher stress-related sick leave, and staff turnover (Bakker and Sanz-Vergel, 2013; Bakker, 2017). In contrast, in operations with higher levels of job resources, such as sense of influence in work or social community, it is more likely that employees experience goal accomplishment, learning, personal growth and development, as well as higher levels of work engagement and job satisfaction (Schaufeli and Bakker, 2004; Bakker and Demerouti, 2008; Bakker, 2011). In line with the basic propositions of the JD-R theory, the present study investigates the significance of specific job demands and resources in relation to employees’ well-being within a religious organization. Furthermore, in this context, we introduce and investigate the role of employees’ credence in the organization’s ability to respond to change.

### Occupational Well-Being – Job Satisfaction and Work Engagement

Employees’ attitudes to work and subjective perceptions of working conditions have been the focus of research in many different disciplines and studied through a vast variety of constructs (Judge et al., 2017). Job satisfaction, as an assessment of workers’ positive or negative attitudes to their job, is seen as a significant indicator of employee well-being (Judge et al., 2017). Furthermore, work engagement is an aspect of employee well-being that refers to vigor, dedication, and absorption (Schaufeli and Bakker, 2004; Bakker, 2011). Even though the two concepts target essential aspects of employee well-being, they are differentiated, as work engagement involves some extent of activeness, whereas job satisfaction is considered to be a more passive state (e.g., Bakker, 2011; Judge et al., 2017). Moreover, employees’ job satisfaction and work engagement are important aspects of well-being and relate to retention (e.g., Freund, 2005; Halbesleben and Wheeler, 2008; Geisler et al., 2019b). In addition, the focus on job satisfaction and work engagement is in line with the positive psychology approach and the framework of healthy organization, by emphasizing the importance of meaning in work and occupational well-being (e.g., Gable and Haidt, 2005; Di Fabio, 2017).

### Occupational Well-Being Within Religious Organizations

The existing evidence suggests that the basic assumption of the JD-R theory could be applied to assess employees’ well-being in a broad variety of organizations (e.g., Schaufeli and Taris, 2014; Lesener et al., 2019). However, it has been noted that specific investigations of employees’ well-being within religious organizations are rather limited (Bickerton et al., 2014: Miner et al., 2015; Roof, 2015; Ariza-Montes et al., 2017; Ariza-Montes et al., 2018). Furthermore, previous research on employee health and well-being within religious organizations has predominately focused on stress and ill health. For instance, it has been suggested that burnout among clergy can be explained by the human service aspects of the job (e.g., Adams et al., 2017). Findings within similar human service occupations, such as health care (Mauno et al., 2017) and social work (Lloyd et al., 2002), suggest that jobs characterized by a “helping nature” are associated with stress and emotional exhaustion due to emotionally demanding interpersonal relations (Dollard et al., 2003).

However, recent reports suggest that in order to understand occupational health and well-being among employees in religious organizations, research needs to address occupation-specific variables such as the unique dimension of faith and the double identities that employees may have vis-à-vis their work (e.g., Innstrand et al., 2011; Bickerton et al., 2014; Frederick et al., 2018). For example, a calling, the experience that one is destined to fulfill the virtues of one’s profession, is a characteristic that has received attention in previous research (e.g., Thompson and Bunderson, 2019). Through their work, in general, and within religious organizations, in particular, employees can experience fulfillment of “a greater purpose,” which provides meaning on a personal and/or a professional level. However, if employees feel that organizational initiatives put the authenticity and meaningfulness of work at risk, this can lead to employee disengagement and cynicism (e.g., Bailey et al., 2017).

Taken together, employees in religious organizations can often be considered to have both a personal (e.g., personal faith, personal calling) and a professional (e.g., being an employee/a representative of the organization) identification with work. The extent that employees within religious organizations experience congruence between the two dimensions of identification can be expected to influence occupational well-being. Thus, in addition to working conditions in terms of demands and resources, it is important to investigate the role of employees’ credence in the organization’s ability to respond to change (e.g., external and societal conformal pressure to change and adapt) in order to understand employees’ well-being in religious organizations.

### The Church of Sweden – Contextual Change and Occupational Well-Being

In the last few decades, the Church of Sweden (Lutheran) has undergone substantial changes. In the year 2000, the formal relationship between the Swedish State and the Church of Sweden ended. As a result, many people decided to end their membership in the Church of Sweden, which led to financial losses for parishes. Not as a consequence of the separation, but as part of a continuous decrease in the number of parishes (roughly from 2500 to 1400), the Swedish National Church Council decided in 2012 to undertake a major organizational change by amalgamating parishes (Hansson and Andersen, 2001; Hansson and Hansson, 2018). This decision was a response to the contextual changes of the institutional conditions for the Swedish church, resulting in a change from smaller parish units to larger organizational units (Hansson and Hansson, 2018).

Current studies of working conditions and well-being among the employees within the Church of Sweden are limited. However, the Swedish Work Environment Authority has conducted workplace inspections (2007), resulting in the reports of work-environment concerns in terms of high levels of stress and a lack of an organizational support for the employees. Furthermore, some studies suggest that employee well-being within the Church of Sweden relates to the extent that personal values are in line with those of the parish. In a study within the Church of Sweden by Hansson et al., ∼18% of the respondents expressed a sense of misfit between their personal values and the values of the congregation. Moreover, respondents who experienced a misfit also reported lack of employer support, inadequate workplace communication, and higher absenteeism (Hansson et al., 2014). More recently, the results of a study by Hansson and Hansson (2018) among union members employed within the Swedish Church showed that although the majority (82%) of the respondents reported that they found their job joyful and stimulating; every other respondent reported that the workload was too high (i.e., 43%) and that they had considered quitting their job during the last few years (53%). In addition, 20% of the women and 12% of the men reported having been absent due to work-related sick leave in the last year. Overall, the findings by Hansson and Hansson (2018) suggest that challenges in well-being relate to lack of goal clarity, discrepancy between work tasks and competence, lack of adequate management, lack of coworkers, and high level of fatigue.

In all, previous reports suggest that working conditions within the Church of Sweden are stressful, where the combination of structural organizational challenges, high demands, and a lack of resources contribute to the explanation of the negative work environment. Furthermore, most previous studies have been based on case studies or qualitative designs, whereas quantitative data of employees’ well-being on the national level are lacking.

### Credence in the Organization’s Ability to Respond to Change

In the context of religious organizations, the role and significance of how employees’ perception of the organization’s ability to cope with challenges relate to employees’ well-being has been overlooked. Based on the consideration that employees in religious organizations both have a personal and a professional identification with work (e.g., Innstrand et al., 2011) and that the (mis)fit between personal and organizational beliefs is important for understanding employees’ well-being (Hansson et al., 2014), this is somewhat surprising. The ability to cope with challenges is at the core of research on the theoretical construct of resilience. In essence, resilience refers to individual’s ability to “bounce back” after dramatic disruptions or disasters (e.g., Kantur and Iseri-Say, 2015; Linnenluecke, 2015; Mallak and Yildiz, 2016; Crowe et al., 2017). Thus, on the individual level, resilience is associated with an employee’s ability to recover and persevere, as well as the capacity to innovate and mobilize strengths in times of adversity. However, research has also attended to organizational resilience, related to the strength of the organization, its ability to recover, and/or its capacity to find innovative solutions to contextual challenges (Linnenluecke, 2015; Mallak and Yildiz, 2016). Furthermore, in the context of occupational health and well-being from an organizational perspective, recent research suggests that organizational resilience is an upstream factor that has an indirect, positive relationship to individual resilience and work engagement through job resources (Taylor et al., 2019).

However, the theoretical conceptualization of the resilience construct is somewhat unclear, as a variety of definitions and measurements are used within and across different research fields. Still, a common denominator is that resilience refers to the ability to cope with change (Linnenluecke, 2015; Crowe et al., 2017). Furthermore, it has been proposed that the core components of organizational resilience pertain to robustness, agility, and integrity (Kantur and Iseri-Say, 2015). When applied in the context of religious organizations, employees’ perceptions of the organization’s capacity to respond to conformal pressure (in ways that protect basic values, is constructive, and supported by its members) basically reflects the extent to which these actions and adjustments comply with the personal values of the employees. Thus, in the present study, assessment of organizational resilience was used to operationalize employees’ credence in the organization’s ability to respond to changes.

### The Present Study

Based on previous research on occupational health and well-being within religious organizations (Hansson, 2006; Bickerton et al., 2014; Hansson et al., 2014; Adams et al., 2017; Hansson and Hansson, 2018; Thompson and Bunderson, 2019), and the theoretical framework of the JD-R model (e.g., Schaufeli and Bakker, 2004; Bakker and Demerouti, 2017, 2018), the aim of the present study was twofold. First, we investigated how specific characteristics of the job, in terms of certain job demands (role conflict and emotional demands) and job resources (influence at work and social community at work), contribute to the explanation of employee well-being (work engagement and job satisfaction) within the Church of Sweden. Second, acknowledging that religious organizations face challenges and a need to respond to contemporary pressure for change, and that organizational response and adjustments to these requirements may in turn influence employees’ attitudes toward work, a further aim of the present study was to examine the extent to which employees’ credence in the organization’s ability to respond to change may be related to well-being.

## Materials and Methods

### Participants and Procedure

The data for the present study was collected as part of a larger workplace survey conducted within the Church of Sweden. Initially, a dialogue between the researchers and representatives of the National Employer Organization of the Church preceded the survey. The National Employer Organization distributed information regarding the survey to all employees by the use of a weekly newsletter. Information was also given separately to Union representatives and a reference group of five employees were asked to give feedback on the questionnaire design. These email addresses were handed to the researchers by the National Employer Organization of the Church of Sweden, and were used for distribution of the link to the survey. The web-based survey was distributed by the researchers by email to a total of 3,652 ministers and deacons to the survey employed within the Church of Sweden. In total, three reminder emails were sent out in a period of 2 months. The survey took about 15–20 min to complete. In all, 2,112 employees answered the survey (58% participation rate: 72% ministers, 28% deacons; 62% women, 37% men, 1% indicated their gender as other; mean age = 52 years, SD age = 9.6 years). Furthermore, the sample was differentiated in terms of professional tenure: <5 years = 48%; >5 years = 52%). The study was approved by the Swedish Ethical Review Board, Regional secretariat in Lund (dnr: 2017/655). Participants received written information about the research project in the email and were provided with a link to the survey. Informed consent was obtained from all participants.

### Materials and Measures

The data were primarily collected by the use of specific scales of the validated Swedish version of the Copenhagen Psychosocial Questionnaire (COPSOQ II: Pejtersen et al., 2010; Berthelsen et al., 2018), pertaining to employees’ perceptions and experiences of their work conditions and work-related well-being. Items on the COPSOQ were rated on 5-point Likert-type scales, and the scores were then converted to the scale 0–100 (i.e., 0, 25, 50, 75, and 100) before the calculations of the mean item score. Respondents who answer less than half of the questions were set as missing (cf. Berthelsen et al., 2017; Geisler et al., 2019a). Furthermore, the data collection included a scale for organizational resilience (Kantur and Iseri-Say, 2015), in order to assess employees’ levels of credence in the organization’s ability to respond to changes. The study also included measures of employees’ occupational well-being, in terms of work engagement (Schaufeli et al., 2017) and job satisfaction (COPSOQ II: Berthelsen et al., 2018).

#### Independent Variable

*Role conflict* (Cronbach’s alpha = 0.79) was assessed by two items from COPSOQ II (Berthelsen et al., 2018; c.f. Cronbach’s alpha = 0.65), rated on 5-point scales from 1 (“to a very low degree”) to 5 (“to a very high degree”). The items were “*Are some things that you do at work accepted by some people but not by others?*” and “*Are contradictory demands placed upon you at work?*”

*Emotional demands* (Cronbach’s alpha = 0.73) were measured by two items from COPSOQ II: Berthelsen et al., 2018; cf. Cronbach’s alpha = 0.80, rated on 5-point scales from 1 (“to a very low degree”) to 5 (“to a very high degree”). The items were “*Is your work emotionally demanding?*” and “*Do you get emotionally involved in your work?*”

*Influence at work* (Cronbach’s alpha = 0.75) was assessed by two items from COPSOQ II: Berthelsen et al., 2018; cf. Cronbach’s alpha = 0.74, rated on 5-point scales from 1 (“to a very low degree”) to 5 (“to a very high degree”). The items were “*Is it possible for you to influence important decisions about your work?*” and “*Can you influence your work?*”

*Social community at work* (Cronbach’s alpha = 0.84) was measured by three items from COPSOQ II: Berthelsen et al., 2018; cf. Cronbach’s alpha = 0.80, rated on 5-point scales from 1 (“never/almost never”) to 5 (“always/very often”). An example item is “*Do you feel part of a community at work?*”

*Credence in the organization’s ability to respond to change* (Cronbach’s alpha = 0.87) was measured by the use of seven out of the nine items on the Organizational Resilience Scale developed by Kantur and Iseri-Say (2015; cf. Cronbach’s alpha = 0.92). The scale consists of the three dimensions: robustness (two items, Cronbach’s alpha = 0.76), agility (three items, Cronbach’s alpha = 0.84), and integrity (two items, Cronbach’s alpha = 0.75). Two items on the robustness dimension were excluded from the present study, as they were considered to be irrelevant in a Swedish setting and in the context of the Church of Sweden. The excluded items were “*shows resistance to the end in order not to lose*” and “*does not give up and continues its path*”). Participants were asked to rate, on 5-point scales ranging from 1 (“disagree”) to 5 (“agree”), the extent that they agreed with each item statement based on the overall instruction “*My organization as a whole (i.e., the Swedish church)*….” Items examples are: “…*stands straight and preserves its position*” (robustness), “…*develops alternatives in order to benefit from negative circumstances”* (agility), and “…*is successful in acting as a whole with all of its employees*” (integrity).

#### Dependent Variables

*Work engagement* (Cronbach’s alpha = 0.84) was measured by the use of the three-item version of the Utrecht Work Engagement Scale (UWES-3: Schaufeli et al., 2017; cf. Cronbach’s alpha = 0.77–0.85). The three items on the UWES-3 assess vigor (“*At my work, I feel bursting with energy*”), dedication (“*I am enthusiastic about my job*”), and absorption (“*I am immersed in my work*”), and are rated on 7-point scales ranging from 0 (“never”) to 6 (“always”).

*Job satisfaction* (Cronbach’s alpha = 0.73) was assessed using three items from COPSOQ II: Berthelsen et al., 2018; cf. Cronbach’s alpha = 0.84, rated on 5-point scales from 1 (“to a very low degree”) to 5 (“to a very high degree”). An example item is “*All things considered, how satisfied are you with the future prospects in your work?*”

### Data Analyses

The data were analyzed by correlation analyses and hierarchical multiple regression using SPSS statistics (ver. 25). The hierarchical multiple regression analyses tested the predictive value of job demands (Step 1: *role conflict*, *emotional demands*), job resources (Step 2: *influence at work*, *social community at work*), and credence in the organization’s ability to respond to change (Step 3) for work engagement and job satisfaction, respectively. Mediation analyses were conducted using the PROCESS macro (vers. 2.16.3) for SPSS. The mediation models tested whether job resources (i.e., influence at work and social community at work) mediate the effect of employees’ credence in the organization’s ability to respond to change in relation to well-being (i.e., work engagement and job satisfaction). In line with conventional recommendations (Zhao et al., 2010), the mediation analyses used a 95% confidence interval (CI) with 5,000 bootstrap, bias corrected (BCa). In addition, the proportion mediated [*P_*M*_* = *ab*/(*ab* + *c*′): Preacher and Kelley, 2011; Miocevic et al., 2018] was calculated as the effect size of the mediating effect in the respective mediation model.

## Results

### Descriptive Statistics and Correlations

Table 1 reports the descriptive statistics and correlations between all variables. As can be seen, participants reported overall moderate levels of experiencing role conflict but rather high levels of emotional demands, influence at work, and social community at work. In addition, moderate levels were observed for credence in the organization’s ability to respond to change, work engagement, and job satisfaction. Regarding the bivariate correlations, significant associations were found between all study variables. Most of the observed associations were in the expected directions. However, low correlations were found between emotional demands and credence in the organization’s ability to respond to change (negative direction), as well as work engagement (positive direction). For the dependent variables (i.e., work engagement and job satisfaction), stronger correlations were found in relation to job resources (i.e., influence at work and social community at work) and credence in the organization’s ability to respond to change.

**TABLE 1:** Descriptive statistics and correlations (*n* = 2,027).

	*M*	*SD*	*Skew*	1	2	3	4	5	6	7
1. Role conflict	41.49	24.58	0.156	−						
2. Emotional demands	70.78	16.62	–0.141	0.202	−					
3. Influence at work	69.46	19.35	–0.522	–0.231	–0.140	−				
4. Social community at work	78.15	16.63	–0.817	–0.379	–0.112	0.388	−			
5. Credence^1^	2.72	0.66	–0.158	–0.205	–0.035	0.245	0.228	−		
6. Work engagement	4.47	0.82	–0.682	–0.143	0.062	0.297	0.289	0.180	−	
7. Job satisfaction	65.45	18.45	–0.541	–0.297	–0.143	0.543	0.445	0.349	0.404	−

*All correlations are significant at *p* < 0.001. ^1^Credence = credence in the organization’s ability to respond to change.*

### Hierarchical Multiple Regression Analyses

Hierarchical multiple regression analyses (Table 2) tested the relationship between job demands, job resources, and credence in the organization’s ability to respond to change to the respective outcomes: work engagement and job satisfaction. Separate analyses were performed for the two dependent variables. Step 1 included job demands (role conflict and emotional demands), whereas job resources (influence at work and social community at work) were added in Step 2. Finally, in Step 3, credence in the organization’s ability to respond to change was added. Of note, potential confounding variables were tested for by inserting *gender* (dummy-coded as 1/2/3: 1 = man; 2 = woman; 3 = other), *age* (continuous variable), *tenure* (1 = <5 years; 2 = >5 years), and *profession* (1 = priests; 2 = deacons) in a separate first step. However, as this step only contributed to 1% of the variance explained, it was not included in the final model.

**TABLE 2:** Hierarchical multiple regression analyses.

	Work engagement (*n* = 1,973)				Job satisfaction (*n* = 1,977)			
	*β*	Δ*R*^2^	*F* change	Adj. *R*^2^	*β*	Δ*R*^2^	*F* change	Adj. *R*^2^
**Step 1**		0.029	30.20**			0.094	105.60**	
Role conflict	–0.030				−0.077**			
Emotional demands	0.124**				−0.043*			
**Step 2**		0.111	130.65**			0.279	452.71**	
Influence at work	0.215**				0.391**			
Social community at work	0.190**				0.219**			
**Step 3**		0.006	14.30**	0.144		0.032	108.53**	0.40
Credence^*a*^	0.082**				0.187**			

Standardized beta weights are from the third step of the regression analyses. ^*a*^Credence = credence in the organization’s ability to respond to change. *p < 0.05, **p < 0.01.

#### Work Engagement

For work engagement, the model (Table 2) provided a limited amount of variance explained (∼15%). Still, each step in the model provided a unique and significant contribution to the variance explained. In the full model, all predictor variables – except for role conflict – provided a significant contribution to the explanation of work engagement among the employees. Looking at the standardized beta coefficients, the strongest predictors in the model were influence at work and social community at work, as well as emotional demands (positive direction) and credence in the organization’s ability to respond to change.

#### Job Satisfaction

The regression analysis for job satisfaction (Table 2) provided a substantial amount of variance explained (40%). Again, when added to the model, each step provided a significant contribution of the variance explained. In the full model, each independent variable provided a significant contribution to the explanation of job satisfaction among the employees. With regard to the standardized beta coefficients in the full model, the strongest predictors in the model were influence at work, social community at work, and credence in the organization’s ability to respond to change.

### Mediation Analyses

Mediation analyses further investigated the interplay between job resources and employees’ credence in the organization’s ability to respond to change, in relation to work engagement and job satisfaction. The rationale for the variables tested in the mediation models was based on the results of the regression analyses, demonstrating that job resources (i.e., influence at work and social community at work) and credence in the organization’s ability to respond to change were the strongest predictors of employee work engagement and job satisfaction. However, although the results of the regression analyses demonstrated that both job resources and credence in the organization’s ability to respond to change were the independent variables most strongly related to work engagement and job satisfaction, the results do not provide any information with regard to the possible interplay of these relations. In line with the notion supported by recent research (e.g., Taylor et al., 2019), job resources (influence at work and social community at work) were inserted as the mediating variables of the association between employees’ credence in the organization’s ability to respond to change (independent variable) and well-being (dependent variables: work engagement and job satisfaction).

#### Work Engagement

**FIGURE 1:**
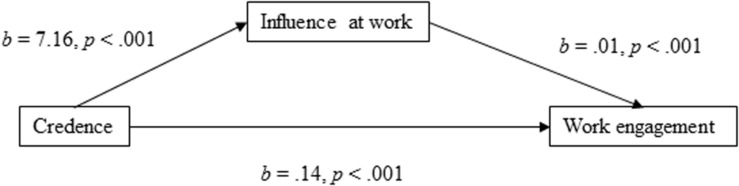
Influence at work as a mediator of the association between credence and work engagement.

For the mediation model of work engagement with influence at work as the mediator (Figure 1), the results showed that the total effect of the model was positively directed [*b* = 0.223, 95% CI (0.164, 0.281), *t* = 7.46, *p* < 0.001]. Credence in the organization’s ability to change had a positive direct effect on work engagement [*b* = 0.140, 95% CI (0.084, 0.197), *t* = 4.87, *p* < 0.001), and the indirect effect of credence through influence at work was found to be significant [*b* = 0.082, 95% CI (0.063, 0.104)]. In terms of the proportion mediated, the effect size of the indirect effect was *P*_*M*_ = 0.37, meaning that 37% of the effect of employees’ credence in the organization’s ability to respond to change on work engagement was mediated by influence at work.

**FIGURE 2:**
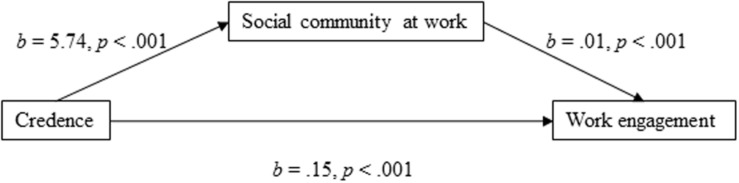
Social community at work as a mediator of the association between credence and work engagement.

For the mediation model of work engagement using social community at work as the mediator (Figure 2), the results showed that the total effect was positively directed [*b* = 0.220, 95% CI (0.162, 0.279), *t* = 7.39, *p* < 0.001]. The direct effect of credence in the organization’s ability to respond to change on work engagement was positively directed [*b* = 0.146, 95% CI (0.090, 0.202), *t* = 5.11, *p* < 0.001], and the indirect effect through social community at work was also found to be significant [*b* = 0.074, 95% CI (0.058, 0.094)]. In terms of the proportion mediated, the effect size of the indirect effect was *P*_*M*_ = 0.34. Thus, 34% of the effect of employees’ credence in the organization’s ability to respond to change on work engagement was mediated through social community at work.

### Job Satisfaction

**FIGURE 3:**
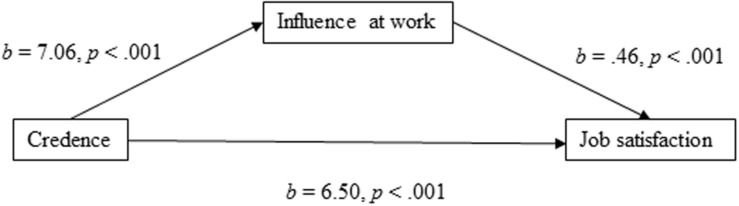
Influence at work as a mediator of the association between credence and job satisfaction.

For the mediation model of job satisfaction with influence at work as the mediator (Figure 3), the results showed that the total effect of the model was evident, significant, and positively directed [*b* = 9.768, 95% CI (8.482, 11.055), *t* = 14.89, *p* < 0.001]. The direct effect of employees’ credence in the organization’s ability to respond to change on job satisfaction was clear [*b* = 6.499, 95% CI (5.422, 7.576), *t* = 11.83, *p* < 0.001], and the indirect effect through influence at work was found to be significant [*b* = 3.270, 95% CI (2.580, 4.001)]. The effect size of the indirect effect was *P*_*M*_ = 0.33, meaning that 33% of the total effect of employees’ credence in the organization’s ability to respond to change on job satisfaction is mediated through influence at work.

**FIGURE 4:**
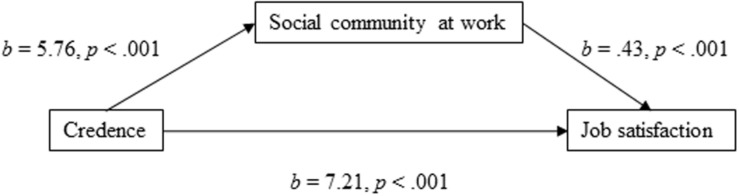
Social community at work as a mediator of the association between credence and job satisfaction.

Finally, the results of the mediation model with social community at work as the mediator (Figure 4) showed that the total effect of the model was clear and positively directed [*b* = 9.654, 95% CI (8.366, 10.943), *t* = 14.69, *p* < 0.001]. The direct effect of credence in the organization’s ability to respond to change was positively directed [*b* = 7.205, 95% CI (5.987, 8.424), *t* = 11.60, *p* < 0.001], and the indirect effect through social community at work was significant [*b* = 2.449, 95% CI (1.955, 3.044)]. The effect size of the indirect effect was *P*_*M*_ = 0.25. In other words, 25% of the effect of employees’ credence in the organization’s ability to respond to change on job satisfaction was mediated through social community at work.

## Discussion

The aim of the present study was to investigate how certain job characteristics, in terms of job demands (role conflict and emotional demands) and job resources (influence at work and social community at work), contribute to the explanation of well-being (work engagement and job satisfaction) among employees within the Church of Sweden. Furthermore, we examined the extent to which employees’ credence in the organization’s ability to respond to changes relates to well-being.

Based on the example of the Church of Sweden, the present study highlights the role of credence in the organization’s ability to respond to change for employee well-being within religious organizations. Overall, the results suggest that credence in the organization’s ability relates to well-being, through an interplay with job resources.

First, the result of the regression analyses showed that our model provided a considerably better explanation for job satisfaction (∼40%) as compared to work engagement (∼15%). As for the predictive value of job demands, in terms of role conflict and emotional demands, these were not found to be determinants of employees’ work engagement. In fact, emotional demands were found to contribute to higher levels of work engagement. This suggests that the emotional aspects of the work may be considered as challenges, contributing to the motivational process (e.g., Lepine et al., 2005; Bakker and Sanz-Vergel, 2013; Geisler et al., 2019a). In some contrast, the results showed that job demands were significantly related (negative direction) to job satisfaction. Second, the results showed that job resources, in terms of influence at work and social community at work, clearly contributed to the explanation of work engagement and, especially, job satisfaction. Third, our results also show that employees’ credence in the organization’s ability to respond to change provided a unique contribution to work engagement and job satisfaction. Although the added contribution was small, the results of the regression analyses demonstrate that credence in the organization’s ability to respond to change is relevant to consider in order to understand employee well-being within religious organizations.

However, the results of the regression analyses do not provide any insights into the interplay between employees’ credence in the organization’s ability to respond to change and job resources in association to occipational well-being. Thus, the mediation analyses were performed in order to further the understanding of the association between credence and job resources in relation to occupational well-being. Overall, the result of the mediation analyses provides support for the notion that the association between credence in the organization’s ability to respond to change and well-being is partly mediated through job resources. The direct and total effects of credence in the organization’s ability to respond to change were more evident with regard to job satisfaction, as compared to work engagement. Still, the effect size of the indirect effects, in terms of the proportion mediated (Miocevic et al., 2018), was found to be clear and rather consistent across the models tested.

### Implications

In terms of implications, the present study contributes to the understanding of well-being among employees within religious organizations (Bickerton et al., 2014; Roof, 2015; Ariza-Montes et al., 2017), by illustrating the role of employees’ perception (credence) of the organization’s ability to meet contextual changes in relation to work engagement and job satisfaction. The focus on occupational well-being among employees in faith-based organizations is much needed, since most previous research has been predominately inclined to attend to stress and ill health (e.g., Innstrand et al., 2011; Adams et al., 2017). Furthermore, our study adds novel insights for the study of employee well-being, by acknowledging that religious organizations are part of society and, as such, exposed to contextual conditions and challenges to adapt and respond to (e.g., DiMaggio and Powell, 1983; Brunsson and Jacobsson, 2010).

Specifically, our results suggest that it is of relevance to attend to employees’ perceptions (i.e., credence) of the extent to which the organization can handle and adaptively respond to change, in order to understand employee well-being. This is in line with the reports by previous research that the organization’s ability to handle change relates to perceived authenticity (Ariza-Montes et al., 2017) and commitment (Elias, 2009) among the employees. To some extent, it may be that the importance of credence is of particular relevance for ocupational well-being of employees within faith-based organizations. This is because employees in religious organizations may be expected to have dual identities toward their work – in terms of a personal (e.g., faith, calling) and a professional (i.e., occupation, member of the organization) identification (e.g., Innstrand et al., 2011; Frederick et al., 2018). However, it may be that the importance of this aspect can be generalized to other types of “identity work” professions (e.g., Brown, 2017). For example, in a wider perspective, calling has been used to explain identification, motivation, and meaning in work across different types of occupational groups such as health care, social work, and law enforcement (e.g., Bakker, 2015; Thompson and Bunderson, 2019).

In conclusion, our study suggest that organizations that are built up on strong values and institutionalized beliefs, such as religious and faith-based organizations like the Church of Sweden, need to tread carefully in the process of adapting to conformal pressure for change, since the actions and choices of the organization are important for employees’ credence in the organization and, in turn, employee well-being. Finally, we encourage future research to further investigate the role of employees’ credence in the organization’s ability to respond to change, in relation to associations and interplays between other job resources, job demands, and outcomes.

### Limitations

The present study has some limitations. The cross-sectional design does not allow any causal claims. Still, the tested directions of the associations seem to be the most plausible ones, and the results therefore provide some insights into the investigated relations and interplays. However, if possible, future research should try to replicate the results and use a longitudinal design in order to test the causal relations. Furthermore, the data are based on self-reports. Hence, the results need to be interpreted with potential common method biases in mind (Podsakoff et al., 2003). Moreover, the present study operationalized employees’ credence in the organization’s ability to respond to change using an existing scale originally designed to measure organizational resilience. To some extent, this could be regarded as a limitation. However, the focus of the present study was to investigate if, and how, employees’ experiences of how the organization can respond to change is important for understanding occupational well-being within religious organizations. Due to the novelty of the present focus, no measure designed to assess this specific aspect exists. The scale used was considered suitable in terms of high face validity. Nevertheless, we encourage future research to continue this investigation, using other scales and measures.

## Data Availability Statement

The datasets generated for this study are available on request to the corresponding author.

## Ethics Statement

The study was approved by the Regional Ethical Review Board, Lund secretariat (dnr: 2017/655). Participants received written information about the research-project in the email and provided with a link to the survey. Informed consent was obtained from all participants.

## Author Contributions

AE participated in the planning of the study, the data collection, the development of the hypotheses, and the data analyses and wrote the first draft of the manuscript. MG contributed to the development of the hypotheses, the data analyses, and the writing of the first draft. TM participated in the planning of the study and the data collection. JB participated in the data collection. HW participated in the planning of the study. All listed authors have made direct and intellectual contributions to the manuscript and approved the final version for publication.

## Conflict of Interest

The authors declare that the research was conducted in the absence of any commercial or financial relationships that could be construed as a potential conflict of interest.
